# Characterization of human corneal stem cells by synchrotron infrared micro-spectroscopy

**Published:** 2007-02-22

**Authors:** Adam J. Bentley, Takahiro Nakamura, Azzedine Hammiche, Hubert M. Pollock, Francis L. Martin, Shigeru Kinoshita, Nigel J. Fullwood

**Affiliations:** 1 Biomedical Sciences Unit, Department of Biological Sciences, Lancaster University, Lancaster, UK; 2 Department of Physics, Lancaster University, Lancaster, UK; 3 Department of Ophthalmology, Kyoto Perfectural University of Medicine, Kyoto, Japan

## Abstract

**Purpose:**

The purpose of this study was to use high resolution synchrotron radiation-based Fourier Transform Infrared (FTIR) micro-spectroscopy coupled with multivariate analysis to investigate the characteristics of adult stem cell (SC) and transit amplifying (TA) cell populations of the human corneal epithelium.

**Methods:**

Spectra of individual SC and TA cells in situ from cryosections of human cornea were collected using a synchrotron micro-spectroscopy facility at Daresbury laboratory, UK. Multivariate analysis and Mann Whitney U tests were used to analyse the spectral data from the SC and TA cell populations.

**Results:**

There were marked differences between the median spectra of the two cell populations. This correlated with their level of differentiation and functional specialization. Multivariate (principal component) analysis revealed that the cell populations could be segregated into distinct clusters, with only slight overlap between the two cell types. Significant (p<0.05) spectral differences were found in the spectral regions associated with nucleic acid, protein and lipids.

**Conclusions:**

Synchrotron FTIR micro-spectroscopy together with principal component analysis is able to discriminate between SC and TA cell populations. Our results also suggest a small sub-population of corneal epithelial cells in the SC niche have TA cell-like characteristics. Many of the spectral differences between the SC and TA cell populations relate to differences in nucleic acid conformation.

## Introduction

**Figure 1:**
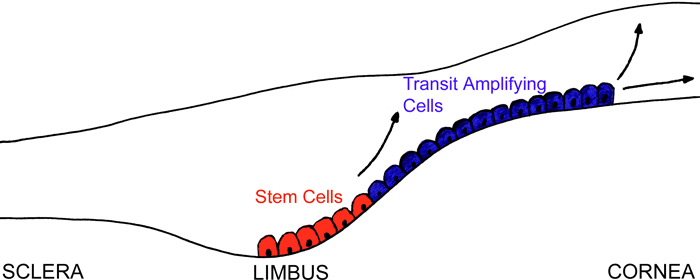
Schematic diagram the corneal epithelium showing the location of the SC and TA cell populations. The stem cells (red) are the basal cell layer located at the limbus; these stem cells proliferate and produce transit amplifying cells (blue) which migrate away from the limbus onto the cornea.

Adult stem cells (SC) are responsible for the regeneration of cell populations in tissues that undergo continuous turnover. These cells are slow-cycling cells with a high capacity for unlimited or prolonged self-renewal extending throughout adult life [1]. One of the better understood SC systems in the body is found in adult corneal epithelium. Davanger and Evensen [2] first hypothesized that the epithelial cells of the limbus may be involved in the renewal of the cornea after observing pigmented cells from the limbus migrate towards the central cornea, as shown in Figure 1. The SC population is believed to be localized to a specific region in the limbus, known as the SC niche [3-5]. Surgical removal of the limbus results in invasion of cells from the conjunctiva [6] whereas grafting of cells from the limbus regenerates corneal like epithelium, further advancing the evidence for corneal SC location.

It is known that corneal SCs can divide either symmetrically to produce two daughter SCs or asymmetrically to produce one daughter SC and one progenitor or transit amplifying (TA) cell. These TA cells, which have only limited proliferative capacity, have been observed to migrate centripetally from the periphery of the cornea forming a basal cell layer. It is also thought that TA cells in turn form terminally-differentiated (TD) cells, which are highly specialized and have no proliferative capacity [7]. The apical layers of the corneal epithelium are made up of these TD cells, which are lost due to desquamation during normal life.

**Figure 2:**
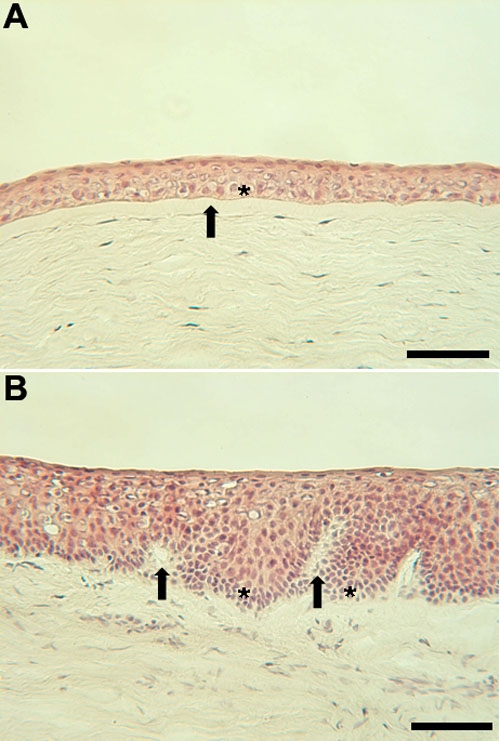
Light micrographs of the corneal and limbal epithelium. **A**: Light micrograph of the corneal epithelium. The TA cells are the basal cell layer (asterisk) below which is Bowman's layer (arrow). **B**: Light micrograph of the limbal epithelium. The invaginations of the basal lamina in the limbal region are termed the palisades of Vogt (arrow). The stem cells are located in the basal cell layer and are concentrated at the base of the rete ridge-like structures (asterisk). The scale bars are equal to 100 μm.

Limbal basal cells are sometimes described as putative SCs, because as yet there is no definitive SC marker. There are, however, a number of molecules which are differentially expressed in comparison with other cells [8]. These so called "markers" include the presence of K5/K14 and p63 and the absence of gap junction proteins [8]. Structurally, SCs possess a more a more primitive appearance (Figure 2B, arrows) compared with the cells of the corneal basal epithelium (Figure 2A).

Studies of the corneal epithelium have contributed enormously to our basic understanding of the way adult SCs function. This is because the SCs here are spatially well-defined and easily accessible for both surgical intervention and observation. It has also facilitated the rapid development of ex vivo SC expansion and transplantation techniques for ocular surface disorders [9-11]. Although a great deal is known about adult human corneal SCs, many questions remain unanswered. The most important of these questions are what determines a SC and, the fundamental differences between a SC and a TA cell.

Advances in high resolution Fourier transform infrared (FTIR) micro-spectroscopy may provide valuable new information on SCs. This powerful technique has been around for almost 40 years but only now is becoming used in cell biology. It has been used to detect subtle intracellular changes associated with diseases such as Alzheimer's disease and osteoporosis, and to discriminate between malignant and non-malignant cells in several different tissues [12-15]. It has demonstrated that it is possible to distinguish cells in different stages of the cell cycle [16] and more recently has shown that it can discriminate between SCs, TA, and TD cells in bovine epithelium [17].

FTIR micro-spectroscopy uses the mid-IR (λ=2-20 μm) absorbance of cellular biomolecules. Each individual chemical bond within the cells gives rise to characteristic spectra. This can provide unique information about structural and conformational changes of the molecules inside the cell [18-21]. Conventional bench-top FTIR spectrometers have a relatively dim thermal IR source, resulting in a much degraded signal-to-noise (S/N) ratio. Consequently single cell resolution measurements are impossible to achieve.

In contrast, synchrotrons provide a highly collimated beam of light that is orders of magnitudes brighter. Using the IR portion of synchrotron radiation, at a spatial resolution of 10 μm, the S/N is about 1,000 times greater [12]. Although the synchrotron radiation source is many times brighter no damage seems to occur to even the most delicate biological samples [22].

FTIR micro-spectroscopy using a bright IR synchrotron radiation source has tremendous potential for the interrogation of intracellular dynamics and molecular changes, e.g., the transition of SCs to TA cells.

FTIR generates massive amounts of complex data, with each individual spectra containing more than 1,000 wavebands. With each experiment requiring the acquisition of tens of spectra, it is often difficult to identify important and usually subtle, differences from the information that is collected. However, recent developments in the use of multivariate analysis and in particular principal component analysis (PCA) now allow more efficient analysis of spectra.

In PCA, each spectrum is assigned a point in n-dimensional space when selected principal components (PCs) are used as coordinates. Cluster analysis of the data is achieved when data plot is viewed in a particular direction. Each of the PCs are eigenvectors of the correlation coefficient matrix of squared deviations. They comprise of a set of new variables, retaining almost all the variation present in the original spectra, with the first PC presenting the most variance, the second PC presenting the maximum amount of the remaining variance and so forth [23]. The information obtained may be plotted in the form of a 3-dimensional plot of the spectral PCs. Briefly, nearness in multivariate distance implies pattern recognition and the separation of sample clusters in the plots signifies structurally dissimilar groups [23].

In this study, we have employed synchrotron radiation-based FTIR (SR-FTIR) micro-spectroscopy to acquire unique in situ information about the putative SC and TA cell populations in human corneal epithelium.

## Methods

### Samples

Corneal specimens were obtained from the Northwest Lions Eye Bank, Seattle, WA. The corneas were all from male Caucasians aged 23, 64, and 66 years. The eyes were harvested within 12 h of death, the corneas placed in Optisol corneal preservation medium (Chiron Vision, CA) and stored at 4 °C for up to 5 days before use. Corneal samples were dissected and frozen. Cryosections (10 μm thick) were collected onto BaF_2_ slides (Photox Optical Systems, Sheffield, UK). The sections were stored in desiccators until required. Parallel sections were stained with hematoxylin and eosin for histological comparison.

### Data collection

Spectral data was collected from cryosections of human cornea at the Daresbury Synchrotron Radiation Source on beamline 11.1 (Daresbury, Warrington, UK) [12]. Spectra were recorded using the Thermo Nicolet Continuum microscope and Nexus FTIR spectrometer. Data were collected in transmission mode and spectra were converted to absorbance using Thermo Omnic software. An area of 10x10 μm was sampled using a 32X Reflachromat objective lens. Spectra were collected at 4 cm^-1^ spectral resolution and co-added for 1,024 scans. Spectra were baseline corrected and normalized to the amide II (about 1,544 cm^-1^) absorbance band using Opus software (Bruker Optics, Coventry, UK). SC spectra were collected from the basal cell layer in the limbal region which consisted of small primitive cells that were poorly differentiated. The location and appearance of these was consistent with that of limbal SCs. TA cell spectra were collected from the basal cell layer in the cornea approximately 3 mm away from the limbus.

### Statistical analysis

Principal Component Analysis was conducted on spectra using the Pirouette software package (Infometrix Inc., Woodinville, WA). After baseline correction and normalization, the spectra were processed as first-derivative (15 points) spectra using Pirouette. Nine PCs were selected for analysis, and loading curves for each PC were plotted for each sample. These loading curves allowed the influence of specific spectral features on each PC to be identified. Score plots (two-dimensional, 2-D) of each PC pair were then plotted for each sample and by combining the clustering evident in these figures with the analysis of the loading curves, the most appropriate 3 PCs were selected for the 3-D cluster analysis. The Mann-Whitney test ("R"; Free Software Foundation Inc., Boston, MA) was employed to compare absorption spectra.

## Results

**Figure 3:**
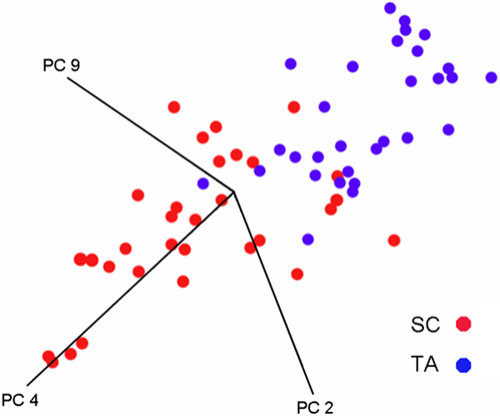
Three dimensional cluster plot of the spectral PCs for SC and TA cell populations. This figure shows a three dimensional cluster plot of the spectral PCs for SCs (red) and TA cells (blue). Nearness of points implies pattern recognition and the separation of sample clusters in the plots signifies structurally dissimilar groups. It is evident that the two populations of cells form distinct clusters, although the discrimination is not perfect.

Figure 3 shows a cluster plot for the principal spectral components for the SC and TA cell populations. It is clear that the two different cell types of interest form discrete clusters. SC and TA cell populations form distinct spatially separate clusters however there is some overlap (about 16%) between these two populations.

**Figure 4:**
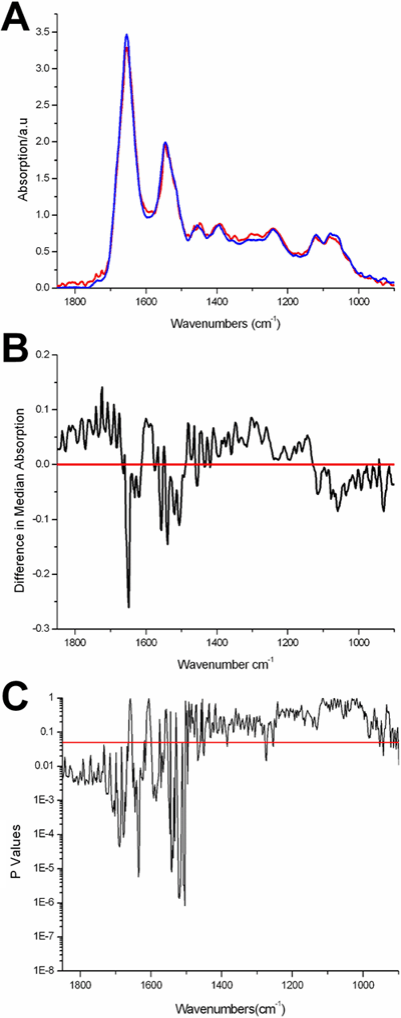
The SC and TA cell median spectra, the difference spectra, and the statistically significant regions of SC and TA cell spectra. Synchrotron FTIR microspectrosopic comparison of SC and TA cell populations in human cornea (**A**). Median spectra for SC (red) and TA (blue) cell populations. **B**: The difference spectra between the SC and TA cell populations. **C**: Significance values for Mann-Whitney comparison of SC and TA spectra (p<0.05 below line).

Figure 4A shows that the median spectra for the SC and TA cell populations appear to exhibit differences, including the regions of the spectrum that primarily relate to changes in nucleic acid conformation [16]. Figure 4B is a difference spectrum (i.e., the difference between the median spectra of the SC and TA populations), which enabled the identification of those regions that exhibited the greatest difference between the cell types. All wavenumbers were subjected to the Mann-Whitney U test and spectral peaks with a significance level of p<0.05 below the red line are indicated in Figure 4C. The positions of many of these regions can be correlated to differences in particular molecular groups. These regions include the area around 1,740 cm^-1^, which is associated with C=O stretching vibrations of lipids [19]. The region around 1,714 cm^-1^ is associated with C=O stretching vibrations of nucleic acids [19]. The region around about 1,650 cm^-1^ is associated with amide I [19]. The region around 1,590 cm^-1^ is related to C=N stretching in guanine [24]. The wave numbers between 1,550-1,500 cm^-1^ are related to vibrational bonds within sugar rings [24]. The region around 1,450 cm^-1^ is due to CH_2_ scissoring in lipids [19]. The peaks around 1,380 cm^-1^ and 1,260 cm^-1^ have been linked to changes in glycoproteins and amide III, respectively [16,18]. Finally, the wavenumbers in the range of 900-950 relate to sugar ring vibrations in nucleic acids [24].

## Discussion

Previous workers have used FTIR micro-spectroscopy with some success in the identification and characterization of many cell types e.g., distinguishing both malignant and pre-malignant changes in several different tissues [12-14] and identifying differences in bovine SC, TA, and TD cells [17]. The purpose of this investigation was to determine whether FTIR microspectroscopy could be used to detect differences between SCs and TA cells in human cornea and shed light on what causes these differences.

The results of our investigation shows that synchrotron-FTIR micro-spectroscopy coupled with multivariate analysis segregates spectra derived from SC and TA cell populations into discrete clusters (Figure 3). This technique shows great potential as a means to discriminate between these different cell types within corneal epithelium. The results observed in Figure 3 show that there is a slight overlap of the SC and TA clusters suggesting that a small proportion, approximately 16%, of the SC population have spectral characteristics similar to TA cells. A possible explanation for this is that there exist a small proportion of TA cells in the SC niche prior to their exit. When a SC divides asymmetrically it produces one daughter SC and one daughter TA cell. Since it must take a significant amount of time for the TA cell to migrate out of the SC niche it is reasonable to expect that a proportion of TA cells might be present in the SC niche at any one time. An alternative explanation is that some SCs are present in the corneal epithelium, but we believe this to be very unlikely as no workers have previously reported finding SCs in this region of the cornea.

Examination of the difference spectrum shown in Figure 4 reveals there are significant differences between the SC and TA cell populations including regions linked to changes in nucleic acids proteins and lipids. It is not surprising that SR-FTIR has detected differences in all these classes of molecules since the primitive undifferentiated SCs have a radically different structure and composition to TA cells. TA cells are functionally more specialized than primitive SCs, and produce a wider range of proteins, which are necessary for specialized function. Such protein types include the keratin pairs K3/K12, clusterin, aldehyde dehydrogenase, and the gap junction protein connexin.

Our finding that SR-FTIR is able to discriminate between SC and TA cells is in agreement with observations made on bovine tissue [17], which also showed statistically significant differences between SC and TA cells. In addition our study also supports previous findings that many of the differences are associated with changes in nucleic acids. This is of considerable interest as it has been reported that the differentiation of SCs is triggered by changes in chromatin structure, which produce favourable conditions for enhancer complexes [25]. Thus these changes in chromatin structure may be contributing to the changes in nucleic acid absorption detected in our study.

To date, FTIR micro-spectroscopy is an under-exploited technique in cell biology. Recent developments mean that it is now possible to obtain spectra from individual cells or even intracellular compartments, and that such spectra can be very conveniently collected from cryosections of tissues. It is even possible now to collect spectra from certain types of live cells [18]. Until recently a major problem was the sheer amount of information obtained even within a single spectrum. However, the availability of software that facilitates rapid multivariate analysis now means that large numbers of spectra within tissues or from different tissues may be routinely compared and that the most significant differences can be pinpointed with a remarkable degree of sensitivity and discrimination.

In conclusion, this study was able to show that synchrotron-FTIR micro-spectroscopy coupled with PCA is capable of discriminating and segregating populations of SC and TA cells in human cornea. Analysis of the different populations suggests that a small subpopulation of cells within the corneal epithelial SC niche appear to possess TA cell-like characteristics, suggesting that these are newly generated TA cells prior to their migration. Analysis also shows that some of the spectral differences are due to changes in nucleic acid conformation. This finding fits in with recent work showing that the control of SC differentiation is related to major changes in chromatin structure [26].
